# Associations between Osteocalcin, Calciotropic Hormones, and Energy Metabolism in a Cohort of Chinese Postmenopausal Women: Peking Vertebral Fracture Study

**DOI:** 10.1155/2021/5585018

**Published:** 2021-03-24

**Authors:** Ruizhi Jiajue, Shuying Liu, Yu Pei, Xuan Qi, Yan Jiang, Qiuping Wang, Wenbo Wang, Xiran Wang, Wei Huang, Xin Zheng, Zhiwei Ning, Ou Wang, Mei Li, Xiaoping Xing, Wei Yu, Ling Xu, Weibo Xia

**Affiliations:** ^1^Department of Endocrinology, Key Laboratory of Endocrinology, National Commission of Health, Peking Union Medical College Hospital, Chinese Academy of Medical Science, No. 1 Shuaifuyuan, Wangfujing Street, Dongcheng District, Beijing 100730, China; ^2^Department of Geriatric Endocrinology, Chinese PLA General Hospital, Beijing 100853, China; ^3^Department of Endocrinology, Beijing Liangxiang Hospital, Beijing 102401, China; ^4^Department Endocrinology, Peking University Shougang Hospital, Beijing 100144, China; ^5^Department of Cadre Unit, General Hospital of the Rocket Force, Beijing 100088, China; ^6^Department of Endocrinology Beijing Haidian Hospital, Beijing 100080, China; ^7^Department of Endocrinology, China Rehabilitation Research Center, Beijing 100068, China; ^8^Department of Endocrinology, Beijing Chaoyang Hospital, Beijing 100020, China; ^9^Department of Radiology, Peking Union Medical College Hospital, Chinese Academy of Medical Sciences, Shuaifuyuan No. 1, Wangfujing, Dongcheng District, Beijing 100730, China; ^10^Department of Gynaecology and Obstetrics, Peking Union Medical College Hospital, Chinese Academy of Medical Science, Beijing 100730, China

## Abstract

**Objective:**

The endocrine function of bone in energy metabolism may be mediated by the osteocalcin (OC). We examined the association between OC and energy metabolism among Chinese postmenopausal women. *Design and Setting*. A cross-sectional cohort study enrolling 1635 participants was conducted using data from the Peking Vertebral Fracture study. Partial correlation analysis was performed to explore the correlation of OC, parathyroid hormone (PTH), or 25-hydroxyvitamin *D* (25(OH)D) with glycemic and lipid metabolic parameters. A logistic regression model was used to investigate the association of OC, PTH, or 25(OH)D with the prevalence of diabetes and dyslipidemia.

**Results:**

Serum levels of OC, PTH, and 25(OH)D were all positively correlated with serum cholesterol levels, whereas only OC was negatively associated with serum glucose level. In the logistic regression model, both OC and PTH were negatively associated with the prevalence of diabetes (odds ratio [OR], 95% confidence interval [95% CI]: 0.967, 0.948–0.986 for OC and 0.986, 0.978–0.994 for PTH). No significant association was found between 25(OH)D and diabetes. Both OC and 25(OH)D, rather than PTH, were associated with abnormalities of high cholesterol levels, such as hypercholesterolemia and high LDL-C levels. Further classifying the population based on the median value of OC and PTH, low OC and low PTH subgroup had the highest OR, 95% CI for diabetes (1.873, 1.287–2.737) and the lowest OR, 95% CI for hypercholesterolemia (0.472, 0.324–0.688) and for high LDL-C (0.538, 0.376–0.771).

**Conclusion:**

Among Chinese postmenopausal women, a lower serum level of OC was associated with a higher prevalence of diabetes and lower serum cholesterol levels, and a low PTH concentration could magnify these associations.

## 1. Introduction

Osteocalcin (OC) is a small noncollagenous protein secreted by the osteoblasts and an indicator of osteoblast activity [1]. In 2007, Lee et al. [2] reported for the first time that OC knockout mice had an impaired beta cell function, decreased insulin secretion and sensitivity, as well as increased visceral fat mass and triglyceride levels. Ever since then, an accumulating body of evidence from animal studies has confirmed the key role of OC in energy metabolism [2–5]. Therefore, bone has been considered as an important endocrine organ modulating energy metabolism, mediated by osteoblast-derived OC.

However, the association between OC and energy metabolism in humans remains to be established, since previous studies showed controversial results. Almost all the cross-sectional studies [1, 6] demonstrated a negative association between OC and the prevalence of diabetes. Nevertheless, prospective studies [7, 8] reported inconsistent results. On the other hand, although the amount of fat mass either in animals [9] or in humans [10, 11] was shown to be inversely associated with OC, the associations between serum lipid profile and OC were conflicting [11–13].

In addition, recent studies have reported close interactions between OC and calciotropic hormones, such as parathyroid hormone (PTH) and 25-hydroxyvitamin *D* (25(OH)D) [14, 15]. Because calciotropic hormones have also been proven to play a crucial role in energy metabolism [16–24], we are curious about whether calciotropic hormones affect the association between OC and energy metabolism. Last but not the least, the associations for these bone-regulating hormones might be largely differed according to gender and ethnics [6, 10, 13, 23, 25].

Therefore, we conducted this cohort study using data from the Peking Vertebral Fracture Study (PK-VF), in order to examine the associations between serum osteocalcin, calciotropic hormones, and energy metabolism among Chinese postmenopausal women.

## 2. Materials and Methods

### 2.1. Subjects

PK-VF is a community-based epidemiologic study designed to determine the prevalence and incidence of vertebral fractures among Chinese postmenopausal women, which has already conducted two surveys in 2008-2009 and 2013-2014 in Beijing, China. A total of 2260 postmenopausal women were enrolled in the second survey. We followed the same methods as Jiajue *R.* et al., published in 2019 [26–28]. Demographic information, years since menopause (YSM), and usage of different drugs were obtained by self-report using a standardized questionnaire answered by each woman. Physical measurements (weight and height) for each participant were performed by certified staff using standard methods. Body mass index (BMI) was calculated according to the acknowledged formula: BMI = weight/height^2^, and women with BMI ≥ 24 kg/m^2^ were defined as overweight or obese. Blood samples were taken by certified staff to perform biochemical analyses, including serum levels of creatinine, alanine aminotransferase, and osteocalcin. In order to investigate the associations between glucose, lipid, bone, and vitamin *D* metabolism among women with natural menopause, we excluded woman coincided with one of these criteria: (1) she still had intermittent menstruation or her menstruation stopped after she took an operation of hysterectomy; (2) she was previously or currently treated with antidiabetic drugs, insulin, lipid-lowering drugs, corticosteroid, anticonvulsant drugs, bisphosphonates, calcitonin, estrogen, or progesterone; (3) she was currently supplemented with vitamin *D* in a daily dose above 10 mcg or supplemented with activated vitamin *D*; (4) her serum level of creatinine was over the upper limit of normal, or her serum level of alanine aminotransferase was higher than two times the upper limit of normal; and (5) her record of osteocalcin was lack. After this exclusion, 1635 women remained and were classified into two groups based on the median level of OC (i.e., OC = 17.11 ng/ml). The study was approved by the Department of Scientific Research, the ethics committee in Peking Union Medical College Hospital (PUMCH). All subjects agreed to participate in this study and signed informed consent forms.

### 2.2. Biochemical Measurements

A fasting blood sample was collected from each woman in the morning (7-9 _AM_). After complete coagulation at room temperature for 30 min, the blood was centrifuged at 2500 g for 10 min and the serum was separated and cryopreserved at −70°C. The serum levels of fasting blood glucose (FBG), creatinine, and alanine aminotransferase were assessed by standard methods in the central laboratory of PUMCH. Serum levels of lipid profile, including total cholesterol (TC), triglyceride (TG), high-density lipoprotein cholesterol (HDL-C), and low-density lipoprotein cholesterol (LDL-C), and serum levels of albumin and glycated albumin (GA) were examined using an automated Beckman spectrophotometry and potentiometry system (AU5821, BECKMAN COULTER Chemistry Analyzer AU5800, US). GA% = GA/albumin. Serum levels of C-terminal telopeptide of type I collagen (*β*-CTX), N-terminal prepeptide of type I procollagen (P1NP), and N-terminal mid-fragment of osteocalcin (N-MID OC), as well as serum levels of calciotropic hormones, including 25(OH)D and PTH (1-84), were measured by an automated Roche electrochemiluminescence system (E170; Roche Diagnostics, Basel, Switzerland). The detection limits of TC, TG, HDL-C, LDL-C, GA%, *ß*-CTX, P1NP, N-MID OC, PTH, and 25(OH)D were 0.10–33.67 mmol/L, 0.00–22.6 mmol/L, 0.052–3.885 mmol/L, 0.026–11.655 mmol/L, 3.2%–68.1%, 0.01–6.00 ng/ml, 5-1200 ng/ml, 0.5–200 ng/ml, 1.2–5000 pg/ml, and 3–70 ng/ml, respectively. The intra-assay and interassay coefficients of variation were 5% and 10% for lipid profile, 3% and 10% for GA%, 2.0% and 4.2% for *ß*-CTX, 2.0% and 2.5% for P1NP, 0.5% and 1.4% for OC, 1.2% and 2.5% for PTH, and 5.2% and 7.5% for 25(OH)D. Reference ranges of these biochemical parameters were obtained from the central laboratory of PUMCH and were all age/sex/ethnic appropriate.

Based on the level in which PTH reached its nadir in relation to 25(OH)D [29], we defined women with 25(OH)*D* < 30 ng/ml as vitamin *D* insufficient and women with 25(OH)*D* < 20 ng/ml as vitamin *D* deficient. Women with serum levels of PTH, TC, TG, and LDL-C over the upper limit of normal (i.e., PTH > 68 pg/ml, TC > 5.70 mmol/L, TG > 1.70 mmo/L, and LDL-C ≥ 3.7 mmo/L) were defined with secondary hyperparathyroidism (SHPT), hypercholesterolemia, hypertriglyceridemia, and high LDL-C, respectively, and women with HDL-C < 0.93 mmo/L were defined with low HDL-C. A woman was considered to have diabetes if she had a clinical diagnosis of diabetes inquired by the questionnaire aforementioned, had a serum level of FBG over 7.0 mmol/L, or had a GA% value beyond the normal reference (i.e., >17.1%).

### 2.3. Statistical Analysis

All the statistical analyses were conducted using SPSS for window version 25.0 (SPSS Inc., Chicago, IL). Data were presented as frequencies (percentages), mean (standard deviation [SD]), median (interquartile ranges [IQR]), correlation coefficient (*r*), odds ratio (OR), and 95% confidence interval (95% CI). The Kolmogorov–Smirnov test was used to verify the normal or skewed distribution of continuous variables. Student's *t*-test was conducted to compare normally distributed continuous variables between women with low OC and with high OC, while Mann–Whitey *U* test was conducted to compare nonparametric continuous variables. Comparisons of categorical variables were performed by Pearson's *χ*^*2*^ test. Partial correlation analysis was conducted to detect the correlations between OC, calciotropic hormones, lipid profile (TG, TC, LDL-C, and HDL-C), and diabetic parameters (FBG and GA%). If a significant correlation was identified, we further adopted a curve estimation to select the best-fitting model. Logistic regression analysis was performed to calculated OR and 95% CI for the status of diabetes and dyslipidemia per SD change in serum levels of OC and calciotropic hormones. *P* value less than 0.05 was considered significant.

## 3. Results

Baseline characteristics of all the participants are summarized in Table 1. Women with low OC levels had a higher prevalence of diabetes than women with high OC levels (69.1% vs. 56.8%, *p* < 0.001). Serum levels of all the BTMs and PTH were suppressed in women with low OC levels, whereas serum level of 25(OH)D was almost the same between groups. Compared to women with high OC levels, serum levels of all the cholesterol parameters (TC, HDL-C, and LDL-C) were decreased and serum level of TG was increased in women with low OC levels. We also conducted Student's *t*-test between diabetics and nondiabetics. Compared to nondiabetics, we found that diabetics have a higher BMI (mean ± SD, 25.76 ± 3.79 kg/m^2^ vs. 25.29 ± 3.70 kg/m^2^, *p*=0.026), lower serum levels of PTH (mean ± SD, 36.40 ± 15.17 pg/ml vs. 39.86 ± 17.81 pg/ml, *p* < 0.001) and OC (mean ± SD, 16.73 ± 7.60 ng/ml vs. 18.67 ± 6.69 ng/ml, *p* < 0.001), and similar serum level of 25(OH)D (mean ± SD, diabetics 15.32 ± 7.19 ng/ml vs. nondiabetics 14.73 ± 7.00 ng/ml, *p* = 0.149).

**Figure 1:**
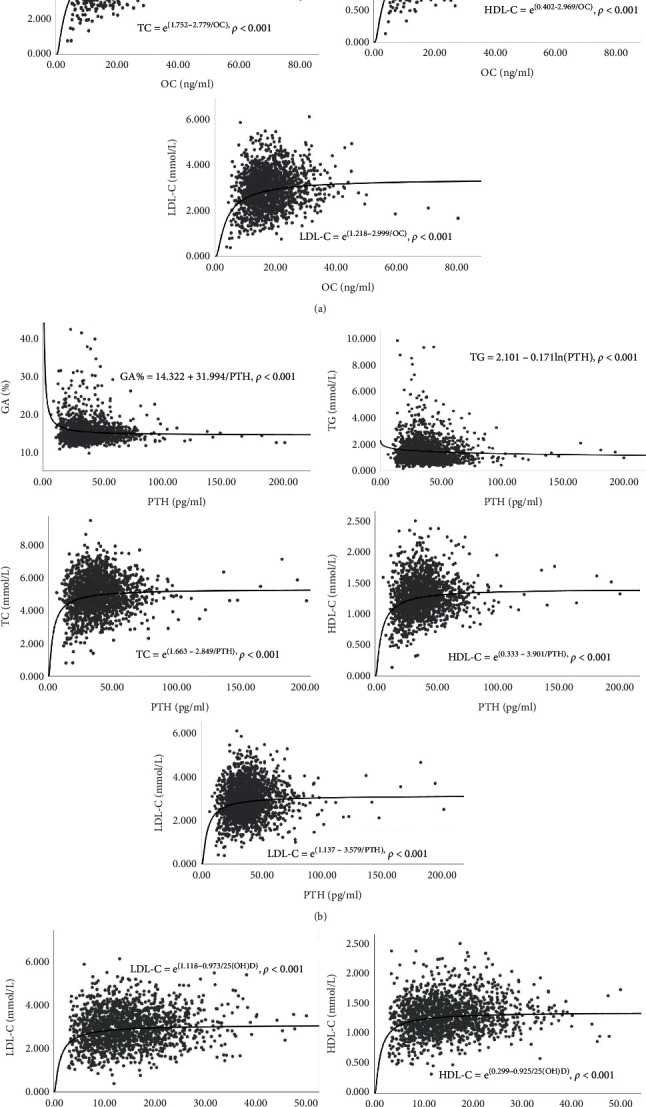
Correlations of parameters of energy metabolism with (A) OC, (B) PTH, and (C) 25(OH)D. FBG, fasting blood glucose; GA, glycated albumin; TC, total cholesterol; TG, triglyceride; HDL-C, high-density lipoprotein cholesterol; LDL-C, low-density lipoprotein cholesterol; OC, osteocalcin; PTH, parathyroid hormone; 25(OH)D, 25-hydroxyvitamin D.

Bivariable correlation analyses were performed first for the associations between age, BMI, 25(OH)D, PTH, and OC. Both age and BMI were positively correlated with PTH (age, *r* = 0.177, *p* < 0.001; BMI, *r* = 0.087, *p* < 0.001), while they were negatively correlated with 25(OH)D (age, *r* = -0.058, *p* < 0.020; BMI, *r* = -0.065, *p* < 0.009). BMI is also negatively correlated with OC (*r* = -0.165, *p* < 0.001). However, the correlation between age and OC is not statistically significant (*r* = -0.012, *p* < 0.631). Further partial correlation analysis revealed that OC was negatively correlated with FBG and GA%, and positively correlated with all the cholesterol parameters, independent of age, YSM, BMI, PTH, and 25(OH)D (Table 2). However, the negative relationship between OC and TG became insignificant after adjusting for PTH and 25(OH)D. Both calciotropic hormones were positively correlated with cholesterol parameters. Besides, PTH also showed a negative correlation with GA% and TG, even with the adjustment of 25(OH)D and OC. No significant correlations were identified between PTH and FBG, between 25(OH)D and glucose parameters, or between 25(OH)D and TC. We must address here that although most of our correlation analyses were statistically significant (*p* < 0.05), the *r* value of less than 0.25 still suggested a poor linear correlation between these parameters. Therefore, we further performed curve estimation to find the best-fitting model (Figure 1).

Logistic regression analysis reported that either increasing OC (OR = 0.967, 95% CI = 0.948–0.986) or increasing PTH (OR = 0.986, 95% CI = 0.978–0.994) was independently associated with decreasing prevalence of diabetes (Table 3). As for dyslipidemia, all the cholesterol parameters were positively associated with both OC and 25(OH)D. No significant association for hypertriglyceridemia was found with all the bone-regulating hormones.

Considering the important roles of OC and PTH, we are wondering whether the associations for OC would differ by PTH. Therefore, we first stratified the population based on the median value of PTH (data not shown) and performed the same logistic regression model among women with different PTH statuses. We found that OC was negatively associated with diabetes only in women with low PTH (OR = 0.957, 95% CI = 0.933–0.982), whereas the positive association between OC and hypercholesterolemia remained significant in both women with low PTH (OR = 1.024, 95% CI = 1.002–1.047) and women with high PTH (OR = 1.049, 95% CI = 1.015–1.084 for hypercholesterolemia). Then, we further reclassified the population into four subgroups based on the median value of OC and PTH (Table 4). Compared to women with high OC and high PTH, women with low OC and low PTH had the highest odds for diabetes and low HDL-C, and the lowest odds for hypercholesterolemia and high LDL-C.

## 4. Discussion

The interaction between bone and energy metabolism has long been investigated. Plenty of studies have indicated the endocrine function of bone in energy metabolism, which is most likely mediated by the BTMs secreted by osteoclast and osteoblast cells [6, 30]. More importantly, recent studies have shown that, among these BTMs, osteocalcin plays the most crucial role [6–8, 12, 31]. Osteocalcin is primarily produced by osteoblasts during bone formation and undergoes posttranslational *γ*-carboxylation with vitamin K as a cofactor. Because *γ*-carboxylation increases the affinity of osteocalcin for hydroxyapatite crystals, the majority of secreted osteocalcin deposits in mineralized bone matrix. Bone resorption processes promote decarboxylation of *γ*-carboxylated osteocalcin (GlaOC) to uncarboxylated osteocalcin (GluOC), decreasing its affinity for hydroxyapatite and therefore promoting its release into the circulation. Therefore, circulating osteocalcin exists in two forms: GlaOC and GluOC. Only the GluOC functions as a hormone to regulate insulin secretion and insulin sensitivity [2]. However, studies measuring different forms of osteocalcin reported inconsistent results about the association between osteocalcin and glucose homeostasis. Total OC was reported to be associated with glucose homeostasis in most studies [10, 11]. As for GluOC, no association with either FBG or insulin resistance was reported in nondiabetic humans [32, 33], while negative associations with FBG and HbA1C were reported in prediabetics [34] and type 2 diabetics [35, 36]. Although GlaOC is considered an inactive component, some studies also reported the inverse association between GlaOC and insulin resistance, which suggested a potential role of GlaOC in glucose homeostasis [32–34]. N-MID OC is the most stable form of OC in serum. In accordance with previous studies investigating N-MID OC [6], our study indeed reported the negative associations between N-MID OC and FBG, GA%, and the prevalence of diabetes. The inverse associations between OC and serum levels of FBG and GA% suggested a modulating function of OC on blood glucose level, which has also been proven by studies investigating FBG [10, 11, 35] and glycosylated hemoglobin (HbAlc) [10, 31, 35]. However, results examining the association between OC and the prevalence of diabetes were controversial [7, 8]. In agreement with our study, almost all the cross-sectional studies [8] have identified a significant association between a lower OC level and a higher prevalence of diabetes, but some prospective studies reported no significant association [7]. The inconsistency may be due to the differences in study subjects and different adjusted confounders.

The underlying mechanisms explaining the protective role of OC against glycemic metabolism and diabetes have been well explored in animal experiments. In OC-deficient mice, insulin secretion is downregulated, glucose tolerance is impaired [2], and a gain-of-function mouse model for OC exhibits an opposite phenotype [2]. Furthermore, administration of OC was shown to improve glucose tolerance and insulin sensitivity in wild-type mice, high-fat diet-fed mice, and diabetic mice [5, 37]. Similar relationships between OC and insulin secretion and glucose intolerance were also demonstrated in human studies [6, 10, 11, 13, 35].

Considering the protective role of OC against diabetes, it is reasonable that OC might also play a role in modulating lipid metabolism. This hypothesis has already been proven with accumulating fat mass found in OC-deficient mice [9]. Furthermore, both Kanazawa et al. [10] and Kindblom et al. [11] found an inverse relationship between osteocalcin and fat mass in human beings. However, scanty studies chose serum lipid profile as their assessment parameter and demonstrated conflicting results [11–13]. Our study reported positive correlations between OC and cholesterol parameters, including TC, HDL-C, and LDL-C, and a negative correlation without statistical significance between OC and TG. More importantly, we found that increasing OC was associated with increased prevalence of hypercholesterolemia and high LDL-C, both of which are well-known risk factors for atherosclerotic cardiovascular disease. Consistent with our results, a negative correlation between OC and TG has also been reported in some studies [11–13]. However, incompatible with our results, Zhou et al. [12] and Ma et al. [13] did not identify any correlation between OC and cholesterol parameters in their Chinese postmenopausal women. And some studies even identified negative correlations between OC and cholesterol parameters [12]. Our findings seem to suggest that OC has a deleterious effect on lipid metabolism, which is contrary to the hypothesis aforementioned. It is unclear why there are such inconsistent results for fat mass and serum lipid parameters. Different selection standards or criteria for the subjects in different studies might be one of the possible explanations, but more studies are needed to clarify the association between OC and lipid metabolism.

More interestingly, we also reported that PTH, independent of OC and 25(OH)D, was negatively associated with the prevalence of diabetes, hypertriglyceridemia, and low HDL-C. Furthermore, comparing to women with higher OC and higher PTH levels, women with lower OC and lower PTH leves had higher risks of diabetes, hyperglyceridemia, and low HDL-C, while had lower risks of hypercholesterolemia and high LDL-C. Therefore, it seems that PTH and OC are independent factors protective against diabetes while promoting cholesterol abnormalities. The similar associations of PTH with lipid metabolism [21, 24] have already been demonstrated in other studies. However, contrary to our findings, most animal studies [17, 20] and human studies [19, 22, 23] investigating the association between PTH and glycemic metabolism showed that it was the evaluated PTH that was associated with glucose intolerance, beta cell dysfunction, and dysglycaemia. Moreover, prospective human studies [19, 23] and studies among patients with primary hyperparathyroidism [16, 18] reported that it was also the evaluated PTH that was associated with increased risk of diabetes. Therefore, PTH is more like a risk factor for diabetes. Considering the cross-sectional design of our study, our results might better be explained by the suppressed bone turnover associated with diabetes [6, 26, 30]. Previous reports found that diabetes impaired the PTH secretion [38], which might be caused by high glucose concentrations [39] or AGEs [40]. Since PTH is known to act on osteoblast [41, 42] and close interactions between PTH and bone formation were identified in our study and others [14, 15], suppressed PTH secretion will suppress bone formation. Nevertheless, it is also worthy of note that Reis JP et al. [23] showed that the association between elevated PTH and incident diabetes varied significantly by race, and PTH injection improved glycemic metabolism in diabetic mice [43]. Considering that most of our study subjects were elderly Chinese postmenopausal women with vitamin *D* insufficiency and normal PTH, there is a chance that suppressed PTH is associated with impaired glycemic metabolism and increased risk of diabetes in this type of population.

This is a large-scale study conducted in a community-based population with high homogeneity. We performed a strict sampling strategy to improve the sample's representation and reduced the potential population stratification, as well as adopting a strict exclusion criterion to eliminate conditions known to affect bone/glucose/lipid metabolism, PTH, and vitamin *D* status. With both bone resorption and formation markers, PTH and 25(OH)D, and different diabetic and lipid parameters examined in every participant, our study guarantees its completeness. The menopausal duration was adjusted for analyses, eliminating its influence on bone metabolism and PTH [44, 45]. The strongest strength of our study is the finding of combined low OC and low PTH associating with higher risks of diabetes and cholesterol abnormalities than low OC or low PTH alone.

There are several inevitable limitations in our study as well. First, the cross-sectional design could only observe the associations, rather than the direction of the associations or causations. Further prospective and mechanistic studies are required to validate these associations. Second, we did not measure the GluOC, which is the metabolic form of OC that actually affects glucose homeostasis [2, 31]. Third, because blood samples were centrifuged and separated before the biochemical examination, red blood cells were discarded, and we could not measure HbAlc. Without HbAlc, we could not detect the influence of OC and PTH on the long-term (3 months) glycemic control and could possibly miss some undiagnosed diabetics. Instead, we measured the GA%, which can also reflect a relatively long-term (2-3 weeks) glycemic control. And the adoption of the FBG and GA% to ascertain diabetes almost eliminate the possibility of missing diabetics.

In conclusion, our study reported that a lower serum level of OC and a lower serum level of PTH were independently associated with a higher prevalence of diabetes and a lower prevalence of cholesterol abnormalities in Chinese postmenopausal women. The vicious cycle between diabetes and OC might be the underlying mechanism for these associations. High blood glucose concentration associated with diabetes suppressed OC secretion directly by suppressing osteoblast or indirectly by suppressing PTH secretion. Decreased OC concentration would then impair insulin secretion and glucose tolerance, resulting in worse glycemic control and increased risk of diabetes.

## Figures and Tables

**Table 1:** Baseline characteristics between groups classified by the median level of OC.

Variables	Reference	Range	Overall	High OC	Low OC	*p*
Number, n	—	—	1635	819	816	—
Age (years), median (IQR)	—	45–93	64.0 (14.0)	63.0 (14.0)	64.0 (15.0)	0.684
YSM (years), median (IQR)	—	1–45	14.0 (16.0)	13.0 (16.0)	14.0 (17.0)	0.634
BMI (kg/m^2^), median (IQR)	—	15.43–37.95	25.15 (4.94)	24.75 (4.88)	25.64 (4.89)	<0.001
Overweight or obese, n/N (%)	≥24 kg/m^2^	—	1029/1635 (62.9%)	465/819 (56.8%)	564/815 (69.1%)	<0.001
Diabetes, n/N (%)	—	—	406/1635 (24.8%)	159/819 (19.4%)	247/816 (30.3%)	<0.001
Diabetic duration (years), median (IQR)	—	0.5–42.0	7.0 (8.1)	6.0 (7.0)	8.0 (9.0)	0.062
FBG (mmol/L), median (IQR)	3.9–6.1	1.90–18.40	5.46 (1.00)	5.37 (0.90)	5.57 (1.16)	<0.001
GA%, median (IQR)	10.8–17.1	9.6–42.0	14.6 (2.3)	14.5 (2.1)	14.7 (2.4)	0.104
TC (mmol/L), mean (SD)	2.85–5.70	0.80–9.42	4.96 (1.05)	5.11 (0.95)	4.82 (1.12)	<0.001
Hypercholesterolemia, n/N (%)	>5.70	—	382/1635 (23.4%)	218/819 (26.6%)	164/816 (20.1%)	0.002
TG (mmol/L), median (IQR)	0.45–1.70	0.20–9.79	1.24 (0.88)	1.21 (0.79)	1.28 (0.96)	0.022
Hypertriglyceridemia	>1.70	—	451/1635 (27.6%)	200/819 (24.4%)	251/816 (30.8%)	0.004
HDL-C (mmol/L), median (IQR)	0.93–1.81	0.14–2.48	1.26 (0.36)	1.30 (0.34)	1.22 (0.36)	<0.001
Low HDL-C, n/N (%)	<0.93	—	164/1635 (10.0%)	40/819 (4.9%)	124/816 (15.2%)	0.001
LDL-C (mmol/L), mean (SD)	<3.37	0.38–6.06	2.91 (1.10)	3.02 (0.79)	2.83 (0.83)	<0.001
High LDL-C, n/N (%)	≥3.37	—	467/1635 (28.6%)	259/819 (31.6%)	208/816 (25.5%)	0.006
*β*-CTX (ng/ml), median (IQR)	0.260–0.512	0.051–1.900	0.412 (0.260)	0.539 (0.238)	0.317 (0.151)	<0.001
P1NP (ng/ml), median (IQR)	15.0–75.0	10.09–249.20	51.88 (25.56)	62.90 (21.58)	40.43 (16.66)	<0.001
OC (ng/ml), median (IQR)	7.46–34.19	3.99–80.60	17.11 (8.05)	21.59 (6.36)	13.54 (4.06)	<0.001
PTH (pg/ml), median (IQR)	12.0–68.0	6.73–200.20	36.44 (18.18)	38.84 (17.76)	33.99 (18.33)	<0.001
SHPT, n/N (%)	>68pg/ml	—	82/1635 (5.0%)	62/819 (7.6%)	20/816 (2.5%)	<0.001
25(OH)D (ng/ml), median (IQR)	8.0–50.0	3.00–50.03	13.72 (9.25)	13.70 (8.91)	13.78 (9.42)	0.742
Vitamin *D* insufficiency, n/N (%)	<20 ng/ml	—	1295/1635 (79.2%)	644/819 (78.6%)	651/816 (79.8%)	0.568
Vitamin *D* deficiency, n/N (%)	<30 ng/ml	—	1583/1635 (96.8%)	794/819 (96.9%)	789/816 (96.7%)	0.768

n, number; SD, standard deviation; IQR, interquartile range; YSM, years since menopause; BMI, body mass index; FBG, fasting blood glucose; GA, glycated albumin; TC, total cholesterol; TG, triglyceride; HDL-C, high-density lipoprotein cholesterol; LDL-C, low-density lipoprotein cholesterol; *ß*-CTX, C-terminal telopeptide of type I collagen; P1NP, N-terminal prepeptide of type I procollagen; OC, osteocalcin; PTH, parathyroid hormone; SHPT, secondary hyperparathyroidism; 25(OH)D, 25-hydroxyvitamin D.

**Table 2:** Correlations between osteocalcin, calciotropic hormones, and glucose/lipid metabolic parameters.

	OC		PTH		25(OH)D	
	*r*	*p*	*r*	*p*	*r*	*p*
FBG	−0.134	<0.001	0.007	0.776	−0.021	0.393
GA%	−0.074	0.003	−0.099	<0.001	0.015	0.544
TC	0.117	<0.001	0.072	0.004	0.164	<0.001
TG	−0.022	0.387	−0.074	0.003	−0.002	0.923
HDL-C	0.106	<0.001	0.149	<0.001	0.167	<0.001
LDL-C	0.106	<0.001	0.063	0.011	0.138	<0.001

In addition to the adjustment with age, YSM, and BMI, additional adjustments were conducted for OC with PTH and 25(OH)D, for PTH with OC and 25(OH)D, and for 25(OH)D with PTH and OC. YSM, years since menopause; BMI, body mass index; FBG, fasting blood glucose; GA, glycated albumin; TC, total cholesterol; TG, triglyceride; HDL-C, high-density lipoprotein cholesterol; LDL-C, low-density lipoprotein cholesterol; OC, osteocalcin; PTH, parathyroid hormone; 25(OH)D, 25-hydroxyvitamin *D*.

**Table 3:** Odds and 95% CI for diabetes and dyslipidemia.

	Diabetes		Hypercholesterolemia		Hypertriglyceridemia		Low HDL-C		High LDL-C	
	OR	95% CI	OR	95% CI	OR	95% CI	OR	95% CI	OR	95% CI
OC	0.967	0.948–0.986	1.021	1.004–1.038	0.985	0.967–1.003	0.932	0.901–0.964	1.020	1.003–1.036
PTH	0.986	0.978–0.994	1.005	0.998–1.012	0.993	0.985–1.001	0.957	0.943–0.972	1.006	0.999–1.012
25(OH)D	1.010	0.994–1.027	1.031	1.014–1.048	1.002	0.986–1.019	0.898	0.870–0.927	1.033	1.017–1.049

In addition to the adjustment with age, YSM, and BMI, additional adjustments were conducted for OC with PTH and 25(OH)D, for PTH with OC and 25(OH)D, and for 25(OH)D with PTH and OC. OR, odds ratio; CI, confidence interval; YSM, years since menopause; BMI, body mass index; HDL-C, high-density lipoprotein cholesterol; LDL-C, low-density lipoprotein cholesterol; OC, osteocalcin; PTH, parathyroid hormone; 25(OH)D, 25-hydroxyvitamin *D*.

**Table 4:** Odds and 95% CI for diabetes and dyslipidemia between subgroups classified by the median levels of PTH and OC.

	Low PTH + high OC			High PTH + low OC			Low PTH + low OC		
	OR	95%CI	*p* value	OR	95%CI	*p* value	OR	95%CI	*p* value
Diabetes	1.485	1.030–2.139	0.034	1.451	0.985–2.136	0.060	1.873	1.282–2.737	0.001
Hypercholesterolemia	0.766	0.550–1.069	0.117	0.652	0.449–0.948	0.025	0.472	0.324–0.688	<0.001
Hypertriglyceridemia	1.381	0.984–1.939	0.062	1.229	0.850–1.778	0.273	1.406	0.977–2.025	0.067
Low HDL-C	2.655	1.373–5.136	0.004	1.936	0.992–3.779	0.053	5.309	2.819–9.998	<0.001
High LDL-C	0.898	0.656–1.228	0.500	0.983	0.695–1.392	0.925	0.538	0.376–0.771	0.001

Reference group: high PTH + high OC. With adjustment for age, YSM, BMI, and 25(OH)D. OR, odds ratio; CI, confidence interval; YSM, years since menopause; BMI, body mass index; HDL-C, high-density lipoprotein cholesterol; LDL-C, low-density lipoprotein cholesterol; OC, osteocalcin; PTH, parathyroid hormone; 25(OH)D, 25-hydroxyvitamin *D*.

## Data Availability

The data used to support the findings of this study are included within the article.
